# Fluoroscopic Placement of Double-Pigtail Ureteral Stents

**DOI:** 10.1155/DTE.7.175

**Published:** 2001

**Authors:** Gregory L. Chen, Demetrius H. Bagley

**Affiliations:** 1 Department of Urology Kaiser Permanente Medical Center Hayward CA USA; 2 Department of Urology Jefferson Medical College Thomas Jefferson University 1025 Walnut Street, Rm 1108 Philadelphia PA 19107-5083 USA

## Abstract

*Purpose*: Double-pigtail ureteral stent is placed cystoscopically after ureteroscopy. We describe a technique for fluoroscopic placement of ureteral stents and demonstrate its use in a non-randomized prospective study.

*Materials and methods*: Double-pigtail stents were placed either fluoroscopically or cystoscopically in 121 consecutive patients. In the fluoroscopic method, the stent was placed over a guide wire using a stent pusher without the use of cystoscopy. Conversely, stents were placed through the working channel of the cystoscope under vision. The procedure, stent length, width, type, method, ureteral dilation, and use of a retrieval string were noted.

*Results*: A wide range of stent sizes were used. The success with fluoroscopic placement of double-pigtail ureteral stents was 100% (89 of 89 cases). No stents migrated or required replacement. Stents were placed after ureteroscopic laser lithotripsy (53/89) and ureteroscopic tumor treatment (22/89). Cystoscopic visualization was used in 32 additional procedures requiring precise control (15 ureteral strictures and nine retrograde endopyelotomy).

*Conclusions*: The fluoroscopic placement of ureteral stents is a safe and simple technique with a very high success rate. We have used cystoscopic placement only after incisional procedures such as retrograde endopyelotomy, stricture or ureterotomy.


*Diagnostic and Therapeutic Endoscopy*, Vol. 7, pp. 175–180
Reprints available directly from the publisher
Photocopying permitted by license only

© 2001 OPA (Overseas Publishers Association) N.V.
Published by license under
the Harwood Academic Publishers imprint,
part of Gordon and Breach Publishing
a member of the Taylor & Francis Group.
All rights reserved.

### Fluoroscopic Placement of Double-Pigtail Ureteral Stents

GREGORY L. CHEN[a] and DEMETRIUS H. BAGLEY[b]*

[a]*Department of Urology, Kaiser Permanente Medical Center, Hayward, CA, USA;* [b]*Department of Urology, Jefferson Medical College, Thomas Jefferson University, 1025 Walnut Street, Rm 1108, Philadelphia, PA 19107-5083, USA*

*(Received 17 July 2001; In final form 20 September 2001)*

*Purpose*: Double-pigtail ureteral stent is placed cystoscopically after ureteroscopy. We describe a technique for fluoroscopic placement of ureteral stents and demonstrate its use in a non-randomized prospective study.

*Materials and methods*: Double-pigtail stents were placed either fluoroscopically or cystoscopically in 121 consecutive patients. In the fluoroscopic method, the stent was placed over a guide wire using a stent pusher without the use of cystoscopy. Conversely, stents were placed through the working channel of the cystoscope under vision. The procedure, stent length, width, type, method, ureteral dilation, and use of a retrieval string were noted.

*Results*: A wide range of stent sizes were used. The success with fluoroscopic placement of double-pigtail ureteral stents was 100% (89 of 89 cases). No stents migrated or required replacement. Stents were placed after ureteroscopic laser lithotripsy (53/89) and ureteroscopic tumor treatment (22/89). Cystoscopic visualization was used in 32 additional procedures requiring precise control (15 ureteral strictures and nine retrograde endopyelotomy).

*Conclusions*: The fluoroscopic placement of ureteral stents is a safe and simple technique with a very high success rate. We have used cystoscopic placement only after incisional procedures such as retrograde endopyelotomy, stricture or ureterotomy.

*Keywords*: Stent; Ureter; Urinary catheterization; Tumor

### INTRODUCTION

The cystoscopic placement of indwelling ureteral catheters and stents has been a common procedure performed by urologists since it was first described by Zimskind *et al.* [1]. Silicone rubber tubes were placed over a wire along side a cystoscope under direct vision, rather than through the scope [2]. Since there was no means to hold the stent in place, migration was a common problem. Finney was the first to publish his experience with a self-retaining silicone double-J ureteral stent in 1978 [3]. This

*Corresponding author. Tel.: +1-215-955-6961. Fax: +1-215-923-1884. E-mail: demetrius.bagley@mail.tju.edu stent gained acceptance in the urologic community and remains in wide use.

A ureteral stent design commonly used today evolved from the one which was first introduced by Mardis and his associates in 1978. They described a 6F single pigtail stent that is placed over a guidewire and coiled in the renal pelvis as the guidewire is withdrawn [4]. This stent had problems with proximal migration up and out of the bladder, making it difficult to retrieve, so a stent with a second distal pigtail was designed [5]. In the urologic literature, stents are routinely placed through the cystoscope. We perform a non-randomized prospective study of fluoroscopic and cystoscopic stent placements to compare the indications for each use, and describe our technique in fluoroscopically placing ureteral stents.

### SURGICAL TECHNIQUE

Intravenous sedation, general or regional anesthesia is administered and the patient is placed in a dorsal lithotomy position with appropriate padding of the extremities on a radiolucent operating room table. Parenteral antibiotics are administered. Flexible or rigid cystoscopy is performed. The ureteral orifice on the side of interest is located, and in cases involving therapeutic ureteroscopic procedures, a heavy duty 0.038 in. floppy-tipped, Teflon-coated stainless steel guide wire* is passed as a safety wire. Under C-arm fluoroscopy, the wire is guided into the upper pole or the renal pelvis beyond the area of pathology. The guide wire is clipped securely to the drapes with a Kelly clamp and left within the ureter during the entire case. Ureteroscopy is performed for various therapeutic indications including laser lithotripsy, endopyelotomy and ablation of upper tract tumors.

After ureteroscopic inspection and treatment is completed, 10 ml of 30% contrast in saline is injected through the working channel of the ureteroscope, an open ended catheter or a double lumen catheter to opacify the intrarenal collecting system. If a safety wire was not used during the case, a guidewire may be passed through the working channel of the ureteroscope prior to withdrawing the scope. First, the tapered end of the stent is passed over the wire and inserted, until its distal end disappears into the urethral meatus. It is essential that the surgical assistant keep the guidewire straight and taut during stent placement, without allowing it to be advanced or withdrawn. Fluoroscopy is used to follow the position of the proximal end as it is advanced into the ureter, and to assure that the wire and stent do not coil in the bladder.

Next, the "pusher" that is supplied with the stent is placed over the wire until it abuts the stent. The pusher with a radio-opaque metal band on the tip is preferable since it can be used as a marker for the distal end of the stent. The pusher is used to advance the proximal end of the stent into the upper pole of the renal pelvis under fluoroscopy. At this point, the guidewire is withdrawn several centimeters until a complete 360-degree coil of stent is seen in the renal pelvis or selected calyx. The pusher can be gently advanced from 1 to 2 cm to form a coil.

If a coil does not form, this may indicate that the stent is not properly positioned to curl. The proximal end of the stent may be positioned in the proximal ureter, or less frequently, the calyx or renal pelvis is not spacious enough to accommodate a coil of stent. In these situations, there is usually enough friction of the wire within the stent to withdraw or advance both as a unit to reposition the stent to obtain the proper pigtail configuration. If the retrieval string is attached, it can be used to pull the stent back.

The fluoroscopic C-arm is simultaneously positioned over the bony pelvis to assure that the distal end of the stent is not coiled in the bladder and has not migrated into the ureter. The marker on the pusher is advanced to the upper edge of the symphysis pubis in a male patient, or lower margin of the symphysis in a female. The guidewire is withdrawn until only a few centimeters are visible in the distal end of the stent. The pusher is advanced several centimeters and a distal pigtail coil will form in the bladder. The C-arm is directed one last time at the renal pelvis to confirm

*Cook Urological Inc., Spencer, IN 47460.

that the proximal pigtail maintained its position. If the distal end of the stent is not curled, it may be in the urethra and can be advanced with the pusher. Care must be taken to prevent placing the distal end of the stent into the ureteral orifice, especially when a retrieval string is not used.

We prefer to use a pushable radio-opaque double-pigtail ureteral stent as opposed to a double-J stent design because the double-pigtail design can be passed easily over a wire and it less commonly migrates. If the stent is to be left in place for less than 2 weeks, a retrieval string may be used and taped to the base of the penis in a male, or pubis in a female for easy removal. When stents are left indwelling for greater than 2 weeks, no string is attached and the stent is removed cystoscopically in the office.

When stents are placed cystoscopically after a ureteroscopic procedure, the guidewire is backloaded through the cystoscope sheath and working channel, and the scope placed through the urethra over the wire. The stent is passed over the wire and the pusher used to advance it up to the distal stent marking at the ureteral orifice. The guidewire is withdrawn to form the proximal and distal pigtail [5]. The proximal pigtail formation is observed under fluoroscopy while the distal pigtail is confirmed with the cystoscope.

### RESULTS

Between September 1998 and May 1999, 121 consecutive patients underwent placement of a ureteral stent. All procedures were performed in the operating room under the supervision of one attending urologist. Ages ranged from 3 to 89 years and included 56 females and 65 males. One hundred eighteen patients underwent an ureteroscopic procedure, and three had a stent placed prior to extracorporeal shock wave lithotripsy (Table I). The ureter was dilated with various instruments prior to stent placement at the end of the procedure (Table II). Most commonly, the ureter was dilated with a 10 Fr double lumen catheter to gain access for ureteroscopy (73/121 cases), followed by dilation with a 6.9 Fr tip semirigid ureteroscope (38/121 cases). Various double-pigtail ureteral stents were used (Table III) with dimension from 4.8 to a reversed 7/14 Fr endopyelotomy stent (Table IV) and length from 14 to 28 cm (Table V). A heavy-duty Teflon coated guidewire was utilized in 104 cases. A hydromer-coated wire was used in 17 cases with silicone stents. The proximal stent curl was placed in eight upper pole calyces, four lower pole calyces, and 109 renal pelvises.

All 89 of 89 attempts at placing stents fluoroscopically were successful (100%). A 6 Fr by 24 cm Percuflex® stent was the most common stent placed in this group (80/89 cases). A retrieval string was used in 67 cases of 89 cases (75%). The most common indication for ureteroscopy was laser lithotripsy and extraction of calculi in the fluoroscopic stent group (53/89 cases), followed by upper tract TCC resection (22/89).

In 32 additional cases, stents were placed initially with cystoscopic visualization without attempting fluoroscopic placement. In these cases, precise control was required or increased ureteral resistance was anticipated. Fifteen of these patients had ureteral

TABLE I Indications for stent placement—procedures and placement technique

| Indication | By fluoroscopy | By cystoscopy |
|---|---|---|
| Ureteroscopic laser lithotripsy and extraction of calculi | 53 | 3 |
| Ureteroscopic biopsy and ablation of renal or ureteral mass | 22 | 3 |
| Stent placement prior to ESWL | 2 | 1 |
| Migrated stent exchange | | 1 |
| Ureteral stricture incision/dilation | 1 | 15 |
| Ureteroscopic endopyelotomy or calyceal diverticulum incision | 6 | 9 |
| Diagnostic ureteroscopy for essential hematuria or colic | 5 | |
| Total | 89 | 32 |

TABLE II Method of ureteral dilation for ureteroscopic procedure prior to stent placement

| Method of ureteral dilation | By fluoroscopy | By cystoscopy |
|---|---|---|
| 10 Fr double lumen catheter | 50 | 23 |
| Semirigid ureteroscopy | 32 | 6 |
| Balloon dilation of the ureter | 2 | |
| 14 Fr Amplatz sheath dilation of ureteral stricture | 1 | |
| 7 Fr open ended catheter | 1 | |
| No dilation required | 3 | 3 |

TABLE III Stent type used

| Stent type | By fluoroscopy | By cystoscopy |
|---|---|---|
| Percuflex® (Microvasive) | 80 | 13 |
| Black silicone (Cook) | 2 | 15 |
| Bard | 2 | |
| Sof-flex® 10 Fr (Cook) | 4 | 3 |
| 7/14 endopyelotomy stent (Microvasive retromax®) | 1 | 1 |

strictures, nine had undergone retrograde endopyelotomy, three had ureteral transitional cell carcinoma, one had a migrated stent changed, and four were treated for stones. Fifteen black silicone stents and 12 Percuflex® stents were placed (Table III). The most common stent size was 7 Fr by 24 cm. None were left with retrieval strings as stents were left for greater than 8 weeks. All 32 of 32 cystoscopic stent placements were successful. There were no complications such as perforations or lost ureteral access in either group.

TABLE IV Width of stents placed

| Stent width (French) | By fluoroscopy | By cystoscopy |
|---|---|---|
| 4.8 | 6 | 1 |
| 6 | 56 | 9 |
| 7 | 17 | 11 |
| 8 | 6 | 5 |
| 10 | 3 | 5 |
| 7/14 | 1 | 1 |

TABLE V Length of stents placed

| Stent length (cm) | By fluoroscopy | By cystoscopy |
|---|---|---|
| 14 | 1 | |
| 16 | 1 | |
| 18 | 3 | |
| 22 | 11 | 3 |
| 24 | 53 | 22 |
| 26 | 15 | 5 |
| 28 | 5 | 2 |

### DISCUSSION

Placement of double-pigtail ureteral stents is one of the most common endourologic procedures performed by urologists. The indications for stent placement have increased with the recent advances in minimally invasive techniques, specifically ureteroscopy and its applications. There are several benefits with placement of a pigtail stent under fluoroscopy rather than cystoscopy. First, a step is saved by not having to backload a wire through the cystoscope sheath and working channel of the cystoscope. Backloading the wire can be an awkward maneuver and the wire could inadvertently be withdrawn. Second, the urethra is not traumatized further with the cystoscope and additional time is not spent reconnecting the light source, camera, and irrigation. The drawbacks are few, except that a learning curve is required until the urologist becomes comfortable with this technique. Several more seconds of fluoroscopic radiation are required to push the distal end of the stent into the bladder, as compared to the standard technique.

As can be seen from our results, we place a stent fluoroscopically whenever possible. We prefer the Percuflex® stent because of its radio-opacity, stiffness, and pushability. It comes with a radio-opaque marker at the pusher tip, which is very helpful to locate the stent–pusher junction when removing the guidewire to curl the distal end of the stent in the bladder. The stent also goes easily over the heavy-duty 0.038 in. Teflon coated guidewire, which we use routinely as a safety and working wire for ureteroscopy. For silicone stents, a hydromer-coated guidewire is necessary to reduce friction between the stent and wire. We utilize a silicone stent if we plan to leave the stent in place for more than a month. Choice of stent width is dictated by the surgical indication. For uncomplicated ureteroscopic stone treatment and tumor biopsy/treatment, the smaller diameter 4.8 or 6 Fr stent with a string is used. It is left in place for only 3–7 days to prevent obstruction from postoperative edema and can be removed easily in the office or by the patient. Stents placed after retrograde endopyelotomy or stricture incisions are larger in size to allow tissue healing around a larger diameter stent. Retrieval strings are not used, to prevent accidental removal of stents that are left in place for 8–10 weeks [6].

Stent length is arbitrarily determined by the patient's approximate height, and by the distance from the renal pelvis to the bladder seen on an intravenous or retrograde pyelogram. Most patients of average size received a 24 cm length stent. Tall patients (6 ft or greater) received a 26 or 28 cm stent. Shorter adult patients (5 ft. or less) had a 22 cm stent placed. In children and patients with pelvic kidneys, 14–18 cm stents were utilized.

The proximal curl of the stent has a tendency to form in the renal pelvis. In situations where a tumor fills the pelvis or where a specific calyx is to be drained, such as after an incision of a diverticular neck, the curl is placed in the desired calyx. Ureteral dilation is not specifically necessary to place a stent, but in this series, most patients had ureteral dilation performed with a 10 Fr double lumen catheter or semirigid ureteroscope as part of the procedure.

In patients with ureteral strictures, small diameter ureters, or situations where excessive friction is encountered, we recommend cystoscopic placement to allow close proximity of the cystoscope sheath to the ureteral orifice. This stabilizes the stent and wire to prevent buckling within the bladder. In this series, a stent was placed cystoscopically in 15 of 16 ureteral stricture cases. Cystoscopic stent placement should also be considered after retrograde endopyelotomy when precise control of the guidewire and stent placement is crucial. In our series, six of 15 endopyelotomy patients had stents placed fluoroscopically, while nine of 15 had stents placed with a cystoscope.

To place a double-pigtail stent successfully with fluoroscopy, several technical points are critical. The stent should pass up the ureter without resistance, especially at the ureteral orifice. This may require ureteral dilation, which is accomplished prior to or during ureteroscopy. The guidewire must be held tightly and not be allowed to advance or withdraw during stent placement. A proximal coil should be confirmed in the intrarenal collecting system with contrast present. To curl the distal end of the stent, the guidewire should be removed when the pusher–stent junction is seen at the top edge of the symphysis in males, and lower edge of the symphysis in females. Fluoroscopy should be used to simultaneously confirm stent position both proximally and distally.

### CONCLUSION

Double-pigtail ureteral stent placement under fluoroscopic guidance is a safe and simple technique that can be performed successfully as an alternative to cystoscopic placement. Procedures such as uncomplicated ureteroscopic stone removal and upper tract tumor treatment are ideal for fluoroscopic placement and the use of a retrieval string. In cases where maintaining access and stent placement is critical, such as retrograde endopyelotomy or after stricture treatment, cystoscopic placement should be given greater consideration.

### *References*

[1] Zimskind, P.D., Fetter, T.R. and Wilkerson, J.L. (1967) "Clinical use of long term indwelling silicone rubber ureteral splints inserted cystoscopically", *J. Urol.* **97**, 840.

[2] Orikasa, S., Tsuji, I., Siba, T. and Ohashi, N. (1973) "A new technique for transurethral insertion of a silicone rubber tube into an obstructed ureter", *J. Urol.* **110**, 184.

[3] Finney, R.P. (1978) "Experience with a new double J ureteral catheter stent", *J. Urol.* **120**, 678.

[4] Hepperlen, T.W., Mardis, H.K. and Kammandel, H. (1978) "Self-retained internal ureteral stents: a new approach", *J. Urol.* **119**, 731.

[5] Mardis, H.K., Hepperlen, T.W. and Kammandel, H. (1979) "Double pigtail ureteral stent", *Urology* **14**, 23.

[6] Conlin, M.J. and Bagley, D.H. (1998) "Ureteroscopic endopyelotomy at a single setting", *J. Urol.* **159**, 727–731.

